# Development and Validation of a Low-Cost Endoscopic Spine Surgery Simulator

**DOI:** 10.7759/cureus.16541

**Published:** 2021-07-21

**Authors:** James K Liu, Paul S Page, Nathaniel P Brooks

**Affiliations:** 1 Neurosurgery, University of Wisconsin, Madison, USA; 2 Neurological Surgery, University of Wisconsin, Madison, USA

**Keywords:** endoscopic brain and spine, spine models, degenerative spine disease, spine microsurgery, neuro spine

## Abstract

Background

Minimally invasive endoscopic techniques in spine surgery continue to gain in popularity. Unfortunately, there is a long learning period for novice endoscope users to acquire basic skills, and complex training simulators are frequently cost-prohibitive. This paper describes the development and validation of a low-cost endoscopic spine training simulator.

Methodology

A low-cost endoscopic spine training model was created utilizing a budget of less than 65 USD. Afterward, a training curriculum consisting of five tasks was designed to mimic standard techniques frequently utilized in endoscopic spine surgery. This curriculum was tested on a cohort of surgical trainees. The initial time to completion as well as errors made during the tasks and repeat trials were recorded. A composite score was generated to quantify the overall scores which included both time and errors in each task.

Results

In total, 11 students and surgical residents completed the curriculum. The first attempt required an average of 622 seconds for the completion of the curriculum compared to 283 seconds in the second trial (p < 0.001; SD = 36.75). In regards to trials in which errors were counted, fewer errors occurred during the second attempt (2.55 vs. 1.53); however, this difference was not statistically significant (p > 0.05). In regards to the composite score, the composite score of the intern group demonstrated an average improvement of 0.345 compared to an average improvement of 0.47 in the resident group.

Conclusions

Our study demonstrates the feasibility of a low-cost endoscopic spine trainer as well as its efficacy in improving basic endoscopic skills in trainees.

## Introduction

Endoscopic spine surgery (ESS) is an increasingly popular approach for spine decompression and discectomy. It was initially described and popularized by Parvis Kambin and then refined by both Mayer and Brock in 1993 and later by Foley and Smith in 1997 [1,2]. Since then, endoscopic techniques have advanced to provide a greater comfort level for spine surgeons attempting to add these procedures to their repertoire or to train residents. Numerous studies have been conducted to evaluate the “learning curve” associated with these procedures. Lee et al. evaluated the first 51 cases of percutaneous endoscopic lumbar discectomy at their institution demonstrating a significantly decreased operative time following the first 17 cases [3]. A larger systematic review conducted of all minimally invasive spine techniques in 2014 demonstrated that the learning curve, as defined by longer procedures times and higher complication rates, typically was overcome after 20 to 30 consecutive cases [4].

Despite the steep learning curve being a barrier to the adoption of these procedures, scant literature exists on methods to reduce this learning curve. Due to the limited amount of literature on this topic, many authors have argued that training simulators may reduce the learning curve associated with these procedures [5-7]. These simulators facilitate a low-stress environment where the surgical trainee is free to gain experience without the potential of harming the patient. While minimally invasive spine simulators have been described and demonstrated with improved times to test completion, these models are frequently costly and difficult to obtain in all settings [7]. Even though few spine trainer models have been shown to decrease actual procedure time, this correlation has been demonstrated in various other endoscopic procedures including endoscopic colonoscopies [8].

Here, we describe and test a low-cost endoscopic simulator using minimal materials and with an easy-to-assemble design. In addition to the development of the model, a basic training curriculum that can be readily and ubiquitously adapted for use by a spine surgery training institution was created to increase familiarity and improve endoscopic surgical skills. This curriculum offers an avenue to establish the basic skills necessary for endoscopic spine procedures including orientation, hand-eye coordination, and fine motor skills. We also performed face and construct validation testing of the simulator [9].

## Materials and methods

An endoscopic box simulator was produced to introduce the necessary skills required for successfully performing an endoscopic lumbar discectomy. This design utilizes easily available materials To optimize availability for training in as many environments as possible (e.g., home, work, resource-poor). As part of the construction, five tasks were created and a curriculum was developed to improve the comfort and skill levels of those new to ESS. Specific simulator skills included dissection of soft tissues, evaluation of accuracy with manipulation, and basic understanding of orientation in various fields of view. For a full description of each of the tasks, the specific skills tested as well as images of the trainer see the Appendices.

Construction of the model utilized a DEPSTECH Wireless Endoscope, WiFi Borescope Inspection 2.0 Megapixels HD Snake Camera (DEPSTECH, Guangdong, China), which was able to be used interchangeably on multiple smartphone or tablet platforms used as the viewing screen (Android or Apple iOS). A razor blade, hot glue gun (or conventional glue), and tape were also used as aids for assembly. The remaining items utilized everyday items including standard cardstock paper, large straw or tube, colored straws, cotton balls, and a cardboard box. Table 1 presents all the materials that were used in construction as well as the current price. In total, the construction cost was approximately 65 USD, not including the Android/iOS viewing device. In reality, the cost of construction can be much lower as our team acquired materials from discarded household items (e.g., using a shoebox in place of the Darice cardboard box).

**Table 1:** Materials used in the development of the box trainer and their associated costs.

Material	Notes	Cost
Apple iPhone, iPad, Android Phone, Tablet		$449-999
DEPSTECH endoscope		$35.99
Darice cardboard box	Dimensions used in testing are 7.5 × 4 × 11 in.	$7.27
Corrugated cardboard sheets	Used to create dividers	$2.99
Colored straws	Preferably jointed	$6.87
Extra-wide drinking straws		$5.99
Narrow marker		~$0
Cotton balls		~$5
Total cost without operating device		$64.11

The primary researcher collected both first and second trials primarily from neurosurgery residents at the author’s home institution. Institutional Review Board approval was sought and this study was deemed to be exempt. The full curriculum included a total of five tasks. A first trial was collected from an attending spinal surgeon with experience in endoscopic procedures for comparison. The time and setting of data collection were kept constant. Participants were directed to perform the tasks sequentially starting with Task 1 with no extended breaks. The time of completion for each task as well as the errors made on Tasks 4 and 5 were recorded. The average time of completion of all participants in the same residency year was computed for each task.

**Figure 1:**
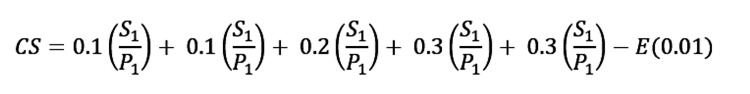
Equation for the calculation of the composite score. S1-5 are the student times for Tasks 1-5, respectively, and P1-5 are the Tasks 1-5 times, respectively, for the corresponding author. E is the number of total errors that were made on Tasks 4 and 5. CS: composite score

In addition to time to completion, a composite score was computed using the algorithm shown in Figure 1, where S1-5 are the student times for Tasks 1-5, respectively, P1-5 are the Tasks 1-5 times, respectively, for the corresponding author, and E is the number of total errors that were made on Tasks 4 and 5.

This training model was created with the intent of reproducing common endoscopic spine techniques used during endoscopic lumbar discectomy. This model was developed at the neurosurgical laboratories of the authors’ institution with the intent of its use and incorporation into the regular schedule spine course for resident education. The model was presented to two neurosurgeons with extensive experience in endoscopic techniques who confirmed its similarity to standard endoscopic lumbar discectomies techniques currently employed. This was used for preliminary evaluation of the face validity, meaning a resemblance to real-life situations based on expert opinions [9].

The ability of the model to actually improve endoscopic skills was assessed via the composite score as described above to access the model’s “construct validity.” The composite score algorithm was created by comparing each of the tasks to a video recording of an in-vivo endoscopic lumbar discectomy and careful consultation with the primary surgeon. Tasks 1 and 2 were given lower weight due to their lower difficulty and their goal of introducing the unique orientation of endoscopy. Tasks 4 and 5 were given the highest weights despite Task 3 being the most similar because they tested advanced fine motor skills expanding upon those tested in Task 3. They were also the highest level of difficulty. Using this process, the performance of novice and experienced surgeons can be compared on the same scale while taking factors such as errors into account.

For a model to be considered valid, the scores should be able to discriminate between novice and experienced surgeons during their initial trials. Content validity is the ability to actually improve the skills being tested. In our study, content validity was assessed using multiple trials and quantified by the composite score, as described above [10]. Statistics were completed using Wizard 1.9.42. The time to completion was assessed using the two-sample t-test. A statistically significant outcome was defined as P < 0.05.

## Results

A total of 11 trainees took part in the study, including eight neurosurgery residents, one plastic surgery resident, one attending physician, and one medical student. The trials were conducted over a four-month period. A total of 10 trainees completed the curriculum once and six trainees completed a second trial with at least a one-week gap between trials.

Of all trainees, the first attempt required an average of 622 seconds for the completion of the curriculum compared to 283 seconds in the second trial (p < 0.001; SD = 36.75). The average improvement in time to completion was 322 seconds. Each individual task ranged from 72.5 seconds to completion on average to 265.5 seconds during the first trial and from 24.8 seconds to 89.2 seconds during the second trial (Table 2). Task 1 and 3 demonstrated statistically significant improvements (p < 0.05). In regards to trials in which errors were counted, fewer errors occurred during the second attempt (2.55 vs. 1.53); however, this difference was not statistically significant (p > 0.05). The average composite score of all participants was 0.22 during the first trial and 0.64 during the second trial.

**Table 2:** Average time to completion in seconds of each individual task.

Average time to completion (seconds)			
	Trial 1	Trial 2	P-value
Task 1	72.5	24.8	0.03
Task 2	84.1	28	0.07
Task 3	265.5	89.2	0.02
Task 4	106	78.7	0.26
Task 5	78.3	62.8	0.42

**Figure 2:**
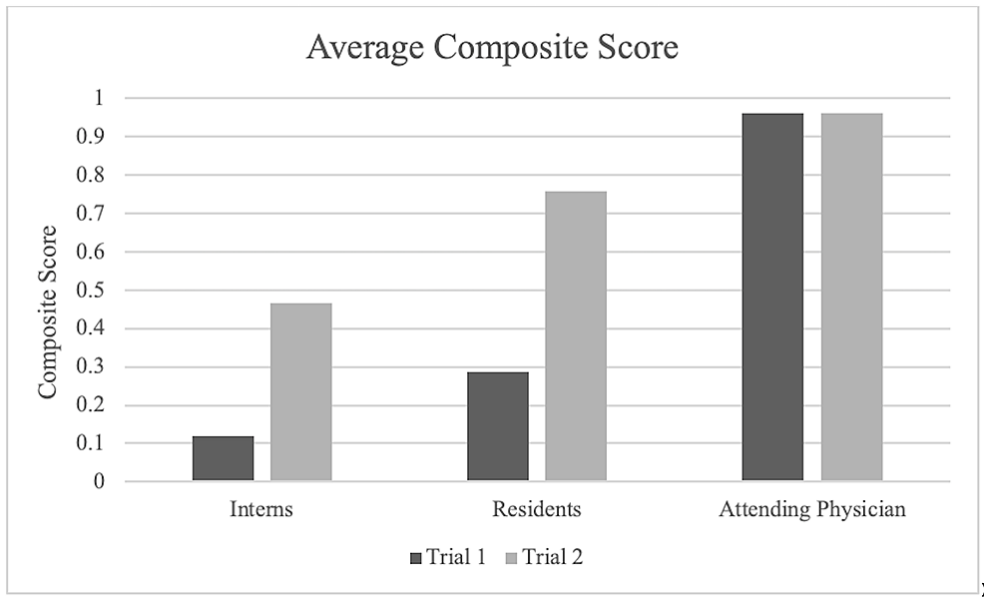
Average composite scores compared with the level of experience.

On further subgroup analysis, interns, residents, and attending physicians were evaluated independently. The composite score was found to be 0.12 in the intern group compared with 0.29 in the resident groups. In both groups, composite scores improved during the second trial to 0.47 and 0.76, respectively. Overall, the composite score of interns demonstrated an average improvement of 0.345 compared to an average improvement of 0.47 in the group of residents who completed both trials (Figure 2). In comparison, the attending physician demonstrated no change and scored 0.98 on both attempts. Interns also demonstrated the greatest average improvement in time to completion of the entire curriculum, that is, a 681-second reduction in time to completion compared to a 177.5 total reduction in the resident group.

## Discussion

Endoscopic lumbar discectomy is a commonly utilized alternative to open lumbar microdiscectomy globally. While open lumbar microdiscectomy has been utilized since the 1970s, more minimally invasive procedures such as endoscopic lumbar discectomy have become more popular due to their ability to be performed under local anesthesia, cause less local tissue disruption, and shorter hospitalization times [11-13]. Despite these improvements, barriers to access exist including the incorporation of newer technologies into practice and the associated long learning curve of using these tools. Of note, the learning curve associated with endoscopic procedures has been well studied and shown to correlate with the reoperation rate and degree of decompression obtained [14,15]. Here, we describe the production, evaluation, and validation of a low-cost endoscopic trainer for percutaneous endoscopic lumbar discectomy.

Endoscopic and other surgical training models are not new and have been utilized in numerous other specialties. Overall, the use of training facilities and simulators in the laboratory setting has been critical in the field of neurosurgery. A recent study surveying 65 neurosurgery residency programs in the United States found that laboratory experience was incorporated into education in 93.8% of programs. While the use of laboratories has been frequently incorporated into these training programs, few programs exist for training in spine surgery. Despite the expanding role of endoscopy in spine surgery, only 15.4% of programs included endoscopic simulators [16]. Despite the benefit of these simulators, the cost can frequently be prohibitive. Given this, we provide these programs as well as other programs internationally with a low-cost option that is readily accessible.

Despite endoscopic procedures having a steep learning curve resulting in reoperations, little research has been conducted regarding reducing this burden [15]. While several studies evaluating this learning curve recommend attending workshops or obtaining microsurgical experience, courses are frequently costly and not always available. Despite the frequent incorporation of simulators in training, currently little to no literature has been published specifically highlighting endoscopic spine simulator training. To date, the only other study in the literature is a recent endoscopic spine model-based lab published by Basil et al. [5]. In their study, cadavers and synthetic models were used to produce an entire curriculum, which was evaluated in the post-lab survey to demonstrate an increase in the comfort level of participants with endoscopic procedures. While this course provides excellent training, these resources are not always available at every institution and require a significant start-up cost.

In regards to validity, numerous surgical training models have become available in recent years; however, standard methods for validating these models are scarce. In our current model, we utilize validation through face validity, content validity, and construct validity. The creation of low-cost stimulators frequently faces issues with face validity, or realism of the simulator to the actual procedure, due to the expense associated with equipment that is frequently used in these procedures as well as the availability of this equipment [9]. Of note, our results demonstrated the ability to differentiate those with less experience from those with more experience, for example, attending physicians, which demonstrates good content validity. The goal of our study was to produce a low-cost trainer with readily available materials globally. While these limitations certainly reduce the realism of the simulator, a high degree of content validity and construct validity exists which improves access where expensive simulators are cost-prohibitive or regionally unavailable.

Our study does contain several limitations. First, the realism and generalizability of our training are certainly not at par with cadaveric models and more expensive simulators; however, as previously stated, this model allows for wide adaptability both locally and internationally. Additionally, our results demonstrated a decrease in time to completion that correlated with experience and repeat trials; however, we were unable to correlate these with operative times or surgical experience. Though our simulator was evaluated by a small number of participants, we believe it does exhibit good content and face validity, thus providing a useful model for entry-level endoscopic spine training. Future studies should further validate this trainer with a comparison of results to cadaveric simulation or surgical times and outcomes.

## Conclusions

We describe the production of a low-cost endoscopic spine stimulator with an associated curriculum. This curriculum and design can be readily utilized in various settings and provide opportunities for areas with minimal access to other training equipment due to its low cost and easy assembly. With further use and implementation, this model can be modified for various procedures and techniques resulting in a huge ability for future expansion.

## Figures and Tables

**Figure 3:**
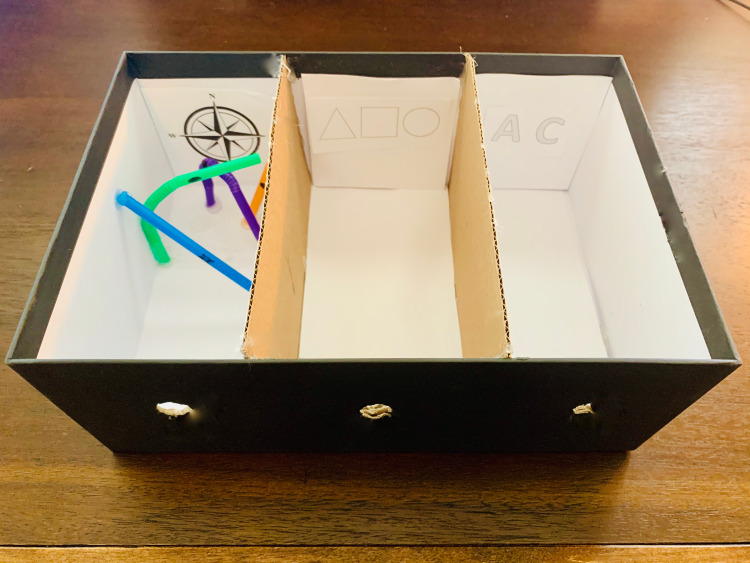
Construction of the endoscopic simulator. Three lanes are created of equal size along with a viewing port anteriorly.

**Figure 4:**
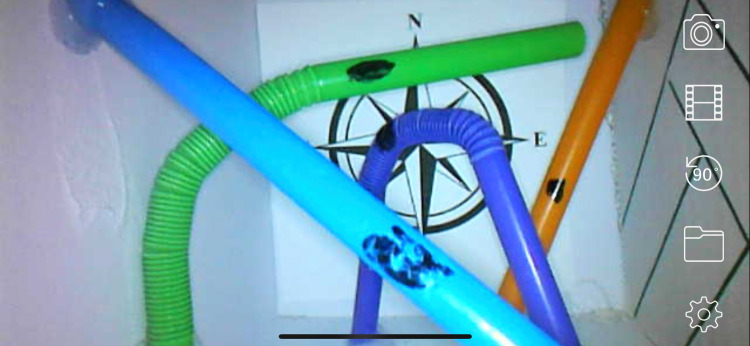
Endoscopic view demonstrating the participants’ view during Task 1. Each straw is easily visualized along with the compass to assist the trainee in understanding the orientation.

**Figure 5:**
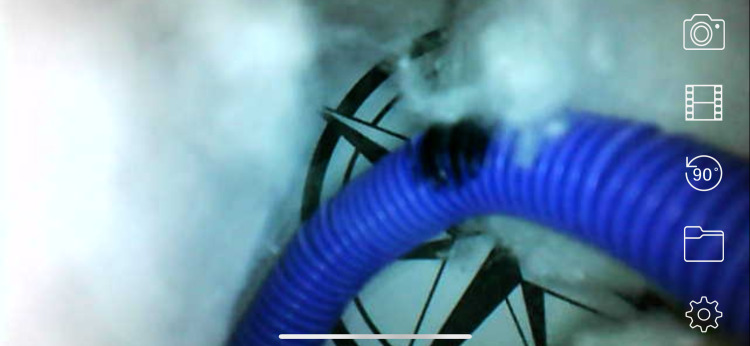
Endoscopic view of the target on the straw after the addition of the cotton balls.

**Figure 6:**
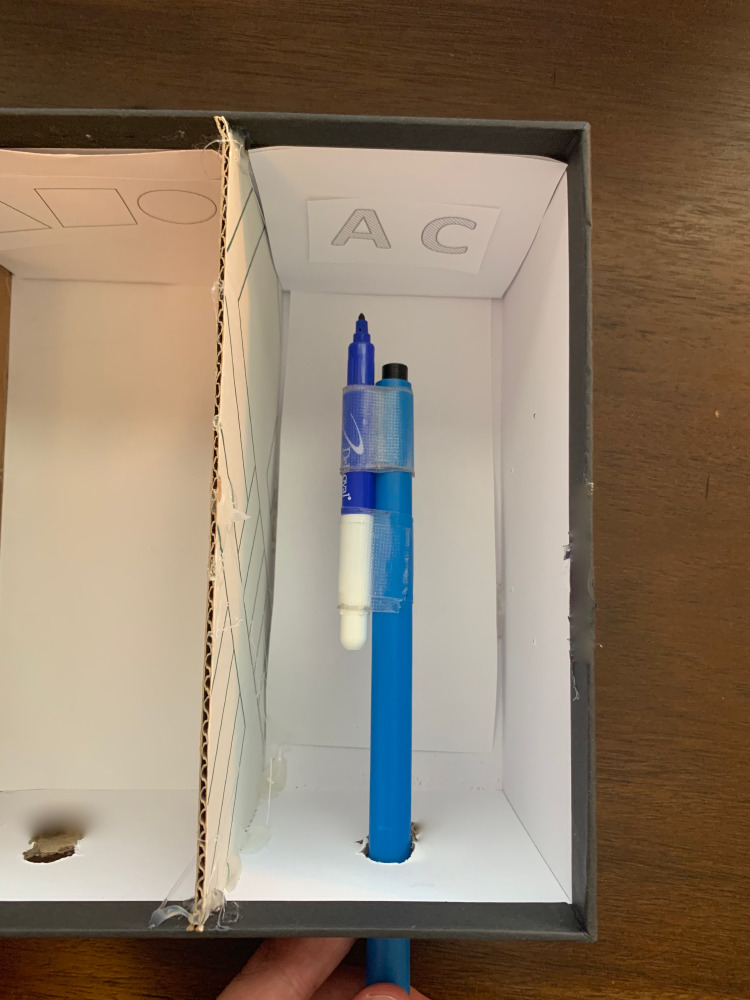
Superior view demonstrating the addition of the marking pen to the working sheath. This view should not be visible to the trainee during task completion.

## Appendices

Construction

The main housing unit of the simulator is created using a 12.5’’ × 9’’ × 5’’ cardboard box or an equivalent size container. In general, any average-sized shoebox can be utilized. Following this, measure three even sections lengthwise on the box of approximately 3.6 inches each. Separate the box into three sections of equal size and cut entryways for the endoscope at the center of each section. Attach cardstock dividers between each section to create three separate length lanes for the tasks described below (Figure 3). In one row, place four straws with a black mark facing the hole where the endoscope will be placed and glue these in place. In this row, a figure containing cardinal directions should be secured to help the examinee orient themselves. The remaining rows should have various shapes and letters placed at the end of the row for the completion of Task 4 and 5. A large smoothie straw or something equivalent should be used to function as a sheath for the endoscope. This will be important to aid in the portions that simulate tissue dissection. A short marker should also be obtained to be attached to the side of the sheath when completing Task 4 and 5.

Task 1: Straws

1.   Use the large beveled straw without any attachment as the sheath.

2.  Orient field of view with help from the surroundings.

3.  Touch each of the straws at the marked black dot.

4.  Time how long this takes.

Goal: Become familiar with the directional movement of the endoscope in relation to the marked directions inside the box. In addition, develop basic hand-eye coordination.

Technical Skills: Orientation, directional awareness, basic endoscope movement, and hand-eye coordination (Figure 4).

Task 2: Cotton Balls

1.   Traverse through the cotton balls by manipulating the endoscope until you reach the back of the box.

2.  Time how long each attempt takes.

Goal: The goal of this lane is to start developing the dissection skills needed in surgical discectomy via a simulated exercise with cotton balls. You will need to get used to how much pressure it takes to disrupt tissue.

Technical skills: Rotational, telescoping dissection, and basic endoscope movement.

Task 3: Straws + Cotton Balls

1.   Use the endoscope to navigate your way through the cotton balls.

2.  There are four straws in this exercise (yellow, blue, green, and pink). Find each one.

Goal: Of all the tasks with this box trainer, this lane most accurately represents a real-life discectomy. It is meant to test the dissection, manipulation, and fine motor skills that will be crucial to successful operations in the future.

Technical Skills: Rotational, angular, telescoping dissection, pressure, orientation, problem-solving, and hand-eye coordination (Figure 5).

Task 4: Shapes

1.   Insert the endoscope using the narrow marker attached to the sheath.

2.  Trace each of the shapes on the backboard about 5 mm inside the printed lines.

3.  Time how long this takes.

4.  Record the number of times that you trace outside of the lines.

Goal: This lane and the associated task is meant to take the motor skills and hand-eye coordination a step further. You will trace a triangle, circle, and square on the back of the box to refine the precision of the endoscope movements and adapt to the presence of a tool that is attached parallel to the view on the scope.

Technical Skills: Hand-eye coordination, precision, and advanced endoscope movement (Figure 6).

Task 5: Letters

1.   Insert the endoscope with the narrow marker attached to the sheath.

2.  Trace each of the shapes on the backboard about 5 mm inside the printed lines.

3.  Time how long this takes.

4.  Record the number of times that you trace outside of the lines.

Goal: There are many times when you are working with an endoscope where very fine movements at the end of a long tool feel very tiring. This task will help train your fine motor movements to tolerate this.

Technical Skills: Fine motor skills and precision with the endoscope, detailed maneuvering of the endoscope, and endurance/conservation of energy.
